# Production Data Set for five-Axis CNC Milling with multiple Changeovers

**DOI:** 10.1038/s41597-025-05294-0

**Published:** 2025-06-23

**Authors:** Mario Martinez, Anna-Maria Schmitt, Andreas Schiffler, Bastian Engelmann

**Affiliations:** Technical University of Applied Sciences Würzburg-Schweinfurt, Institute of Digital Engineering (IDEE), Schweinfurt, 97421 Germany

**Keywords:** Mechanical engineering, Scientific data, Research data, Computer science

## Abstract

This data descriptor contains information about an extensive production data set for a five-axis CNC milling process. Three geometrically different products were manufactured and relevant features from the numerical control of the machine were recorded. The recorded manufacturing process contains the preparation of the machine for the next product (changeover) as well as the machining process (production). The experimental manufacturing was organized with the aid of a changeover matrix to ensure that all possible changeover combinations for the three products were considered. The production was repeated five times, resulting in 30 manufacturing sessions and five complete changeover matrices. The data set was recorded in a laboratory environment. A rich feature set including i.e. the NC-code of the products, tool information, and a Jupyter notebook is provided with the data set.

## Background & Summary

In times of the fourth industrial revolution (Industry 4.0), complete and transparent data are proving to be decisive factors in terms of increasing efficiency in production processes for industrial manufacturing companies^1^. Manufacturing-related data, especially for specific processes such as milling, is scarce compared to the large number of other data sets in repositories^2^. For this reason, this data descriptor and the corresponding data set^3^ also aim to make more production data available for research purposes.

**Fig. 1:**
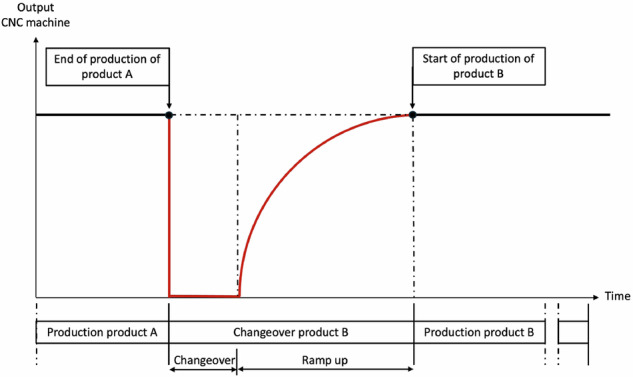
Visualization of changeover and production according to McIntosh *et al*.^19^.

The data was recorded in March 2024 during three days in the machine tool laboratory of the Technical University of Applied Sciences Würzburg-Schweinfurt. The data set^3^ originates from an experimental production of a five-axis milling machine tool. An industrial production using a machine tool not only consists of the machining process in which the part is geometrically created. In the context of production and manufacturing technology, the term ‘changeover’ describes all the activities necessary to set up a machine or production system for a subsequent production order and return it to its original state^4^. The changeover process includes all preparatory and follow-up activities, including dismantling previous tools and devices, installation and setup of new tools, performance of test runs, fine adjustments, and cleaning activities (Fig. 1). The changeover is usually conducted by an operator interacting with the manufacturing machines. During this interaction, several specific time components can be identified, such as the changeover basic time or changeover rest time^5^ (S. 1642). The recorded data set^3^ covers the entire production process, which includes changeover and machining.

**Fig. 2:**
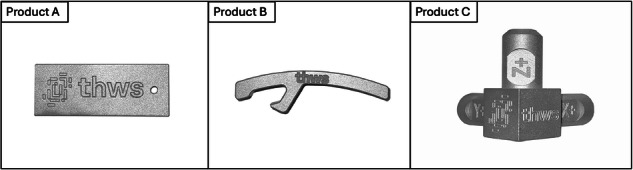
Chosen products for the data set^3^: keychain (Product A), a bottle opener (Product B), and a coordinate system representation (Product C).

For experimental production, three products were chosen to record changeover and production activities. The three products are shown in Fig. 2: a keychain (Product A), a bottle opener (Product B), and a coordinate system representation (Product C). They all differ in the tools used, the changeover time, and the production time. The underlying NC-code for the machining process as well as specifications for tools are provided as external material^6^ to the data set^3^. The experimental production was carried out in a laboratory environment of the university by laboratory engineers.

During previous research, a first data set^7^ was published^2^. The focus of this data set^7^ was to publish data from manufacturing under real-world conditions in a running industrial two-shift production. During thirteen manufacturing sessions, manufacturing data from 13 different products was recorded. Manufacturing was related to existing customer orders that were not repeated. Production was carried out by trained operators from company personnel. The number of features was limited to 20 by a feature selection approach, as well as the number of labels for production phases was limited to three to facilitate the usability of the data set^7^.

To overcome the limitations mentioned, a new data set^3^ was prepared, described in this article. Table 1 compares the previously published data set^7^ (left) with the new data set^3^ (right). In the new data set^3^, all three products were manufactured six times, including machining and changeover sequences. This results in 30 manufacturing sessions exceeding the 13 sessions of the former data set^7^. From the available feature set, only features that showed no information content were excluded from the feature set to allow researchers to design their feature selection approach from the 170 available features of the data set^3^. While in the former data set^7^ the labels for the different phases in the changeover and production process were divided into 2, 6, and 23 phases, in this data set^3^ the process offers many different label sets with up to 73 possible defined sub-phases. The label sets and corresponding sub-phases were derived from a detailed description of the changeover process, available as external material on GitHub^6^. In addition to the changeover process, information about the machining process is also available through the NC-code provided for the three products.

**Table 1:** Comparison of data sets: existing data set^7^ (left), new data set^3^ (right).

Data set name	A Series Production Data Set for Five-Axis CNC Milling	Production Data Set for Five-Axis CNC Milling with Multiple Changeovers
Manufacturing technology	Five-Axis CNC Milling	Five-Axis CNC Milling
Numerical control of machine	HEIDENHAIN iTNC 530	Siemens 840D-SL
Environment Products	Industrial series production with real customer orders under two shift production conditions	Series production in laboratory with three predefined products
Operator skills	Experienced machine operator	Laboratory engineers
Number of manufacturing sessions	13	30
Number of repetitions of sessions	No repetitions	6 manufacturing orders, each repeated 5 times
Number of features	20	170
Number of label sets	3	10
Availability	10.5281/zenodo.10853254	10.5281/zenodo.14094887
License	CC BY 4.0	CC BY 4.0
Additional resources	None	Demo machine learning use case, NC-code and process description, information on applied machining tools

The presented data set^3^ can be used to model technical relationships of the production process between the recorded 170 features. As the changeover phase of production is rich of activities from the human operator in relation with the manufacturing machine, the data set can be used to model human machine interactions. Here, the various label sets for different production phases can be beneficial, as well as detailed information on the changeover procedure itself. The Section ‘Application examples’ portrays two corresponding examples from the research work of the authors.

## Methods

The concept of a changeover matrix is explained in the next subsection. In the subsequent sections, the data collection process, data pre-processing, and labeling are described.

### Concept of changeover matrix

To understand the structure of the data set^3^, it is important to understand the concept of a changeover matrix. A changeover matrix represents all the changeover processes of a machine and their corresponding mean actual changeover times in a transparent form. The changeover matrix shown in Table 2 is an example of the changeover times between three different products of a fictitious machine, with the times given in minutes. The changeover matrix maps all possible setup sequences from one product to the next. The values on the main diagonal are normally zero as no setup process is required for the same product. For better clarity, these entries are marked with an X. The other values in the matrix represent the actual changeover times required to change the machine from one product to another. For example, changing from product A to product B takes 200 minutes, while changing from product B to product C takes 175 minutes. This form of representation makes it possible to quickly identify changeover operations with short or long changeover times, and thus make targeted decisions on which changeover operations should be prioritized or avoided in production planning. In Enterprise Resource Planning software (ERP) SAP, the setup matrix is used to precisely model sequence-dependent setup times and costs, allowing the optimal sequence of production operations to be determined and production operations to be designed more efficiently^8^.

**Table 2:** Example of a changeover matrix for three products with exemplary mean values for changeover duration in minutes.

	

The underlying experimental production of this data set^3^ is organized according to the changeover matrix as shown in Table 2. Three products are produced. However, production was organized so that all six possible changeover combinations between the three products were recorded and a complete changeover matrix was established. This sequence was repeated five times for statistical reasons and resulted in 30 manufacturing sessions with five complete changeover matrices.

### Data collection process

The used five-axis milling machine tool ‘Spinner U5-620’ was built in 2016 and is equipped with a Siemens 840D-SL control. This machine is designed for producing precision parts in various industrial sectors such as aerospace, automotive, and toolmaking. The machine has five axes, including three linear axes, and two rotary axes. The travel paths of the linear axes and the swivel ranges of the rotary axes of the rotary/tilting table are described in detail as follows^9^ (p. 7-96):

- X-axis (linear axis): Max. movement 620 mm from -365 mm to + 255 mm
- Y-axis (linear axis): Max. movement 520 mm from -296 mm to + 224 mm
- Z-axis (linear axis): Max. movement 460 mm from 150 mm above table to 610 mm above table
- B-axis (rotation axis): Swivel range 200^∘^ from -90^∘^ to + 110^∘^
- C-axis (rotation axis): Enables full 360^∘^ rotation

The data from the machine’s numerical control (NC) is acquired by a ‘uaGate 840D’ interface from Softing which was integrated into the machine’s electrical cabinet. The ‘uaGate 840D’ collects data from both the numerical control kernel (NCK) and the programmable logic controller (PLC) using the SIMATIC S7 protocol. A subset of the available features was selected to be transmitted to a database. The selection was based on the features of the previous research, which was expanded to include tool magazine, axis, and drive information. The data was sent in an interval of 1s only if the value changed.

**Fig. 3:**
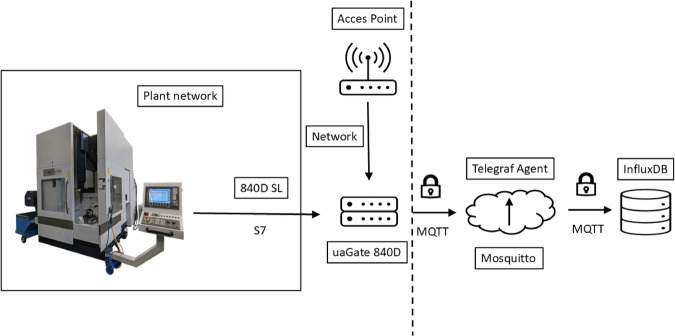
Communication architecture for data acquisition.

As shown in Fig. 3, the gateway acts as an MQTT publisher that forwards the machine data received from the controller to the Mosquitto MQTT broker. The Mosquitto broker receives the messages sent by the gateway and plays a central role in the communication setup by making the received data available to the subscribers. In the present use case, the data is collected and processed by the Telegraf Agent application, which acts as an MQTT subscriber, and then stored in an InfluxDB database.

### Data pre-processing

The recorded data was exported from the database as a CSV file. Data pre-processing begins by filling the first row of all files so that the information of all features is complete. Some features might have missing values, for instance, when no tool is currently in the spindle or when no NC-code line is being executed. These NaN values were retained while missing entries were filled with the last valid value for other features. Subsequently, the data was labeled using the timestamps from the CSV files and the manual recording. A total of 10 distinct labels are identified, as detailed in Tables 3, 4, 5, and 6. Table 3 lists the different label sets. The Tables 4, 5, and 6 show the class numbers and description for all the label sets.

**Table 3:** Label set description.

Label set	No. of phases	Description
Label_01	73	73 different phases observed during production and changeover
Label_02	3	Currently produced product
Label_03	2	Production and changeover
Label_04	4	Same as Label_03, but changeover is divided into start, mid, and end phase
Label_05	6	Combines Label_02 and Label_03
Label_06	4	Extends Label_03 by interruption phases during changeover and production
Label_07	3	Interruption phases from Label_06 are combined
Label_08	12	Distinguishes Label_02 into which product is produced now and was produced before
Label_09	7	Production phases of Label_08 are combined into one
Label_10	43	Phases from the changeover standard, combines phases from Label_01

**Table 4:** Label sets 01, 10, 04 and 03.

Label_01	Description	Label_10	Label_04	Description	Label_03	Description
1	Changeover start - Open door	1	1	Start-Phase Changeover	0	Changeover
2	Removing finished part from previous product	2				
3	Cleaning the working area of the milling machine	3				
4	Dismantling the clamping device	4				
5	Remove the clamping device of the previous product from the machine table	5				
6	Further cleaning of the machine table	6				
7	Moving the clamping device of the new product to the machine table	7				
8	Attaching clamping device to milling machine	8				
9	Go to workbench with tool, insert tool into tool holder	9				
10	Mounting the tool with tool holder in the measuring device and measure	10				
11	Go to the milling machine with tool, tool holder and printout	11				
12	Close door	12				
13	Change tool of previous product in spindle tool holder	13				
14	Open door	14				
15	Remove tool from spindle tool holder	15				
16	Close door	16				
17	Change tool of previous product in spindle tool holder	13				
18	Open door	14				
19	Remove tool from spindle tool holder	15				
20	Close door	16				
21	Change tool of previous product in spindle tool holder	13				
22	Open door	14				
23	Remove tool from spindle tool holder	15				
24	Close door	16				
25	Change tool of previous product in spindle tool holder	13				
26	Open door	14				
27	Remove tool from spindle tool holder	15				
28	Close door	16				
29	Delay/waiting time until work step store tool	41				
30	Open door	17				
31	Insert tool into spindle tool holder	18				
32	Close door	19				
33	Option 1: Enter tool information manually on the control panel	20				
	Option 2: Load tool information					
34	Open door	17				
35	Insert tool into spindle tool holder	18				
36	Close door	19				
37	Option 1: Enter tool information manually on the control panel	20				
	Option 2: Load tool information					
38	Open door	17				
39	Insert tool into spindle tool holder	18				
40	Close door	19				
41	Option 1: Enter tool information manually on the control panel	20				
	Option 2: Load tool information					
42	Open door	17				
43	Insert tool into spindle tool holder	18				
44	Close door	19				
45	Option 1: Enter tool information manually on the control panel	20				
	Option 2: Load tool information					
46	Open door	21				
47	Clamp raw part of new product into clamping device	22				
48	Close door	23				
49	Safe operating mode 2 (DIN EN ISO 16090 chapter 3.4.5)	24	2	Main-Phase Changeover		
	Set zero point (x, y, z)					
50	Unplanned interruption - 01	41				
51	Insert USB stick - select and load machine program	25				
52	Unplanned interruption	41				
53	Safe operating mode 1 (DIN EN ISO 16090 chapter 3.4.4)	26				
	(Execute and optimize the NC program) Execution of machining					
54	Unplanned interruption - 02	41				
55	Unplanned interruption - 03	41				
56	Unplanned interruption - 04	41				
57	End of machining workpiece 1	27				
58	Delay in workpiece removal	41				
	Open door	42	3	End-Phase Changeover		
59	Remove workpiece 1 from the milling machine					
	Measure workpiece 1					
	Clamp new raw part					
	close door					
60	Make further settings on the milling machine 01	31				
61	Make further settings on the milling machine 02	32				
62	No. 2 Safe operating mode 1 (DIN EN ISO 16090 chapter 3.4.4)	33				
	(Execute optimize the NC program) Execution of machining					
63	Unplanned interruption - 05	41				
64	Unplanned interruption - 06	41				
65	Unplanned interruption - 07	41				
66	End of machining workpiece 2	34				
67	Open door	43				
	Measure the workpiece					
	Clamp new raw part					
	Close door					
68	Make further settings on the milling machine 01	38				
69	Make further settings on the milling machine 02	39				
70	Production	40	4	Production	1	Production
71	Unplanned interruption - 08	41				
72	Unplanned interruption - 09	41				
73	Unplanned interruption - 10 - Delay until tool change	41

**Table 5:** Label sets 02, 03, 05, 08 and 09.

Label_02	Label_03	Label_05	Label_08	Label_09	Description
1	0	10	1	0	Changeover - B to A
			3	1	Changeover - C to A
2		20	5	2	Changeover - A to B
			7	3	Changeover - C to B
3		30	9	4	Changeover - A to C
			11	5	Changeover - B to C
1	1	11	2	6	Production - B to A
			4		Production - C to A
2		21	6		Production - A to B
			8		Production - C to B
3		31	10		Production - A to C
			12		Production - B to C

**Table 6:** Label sets 06 and 07.

Label_06	Label_07	Description
10	0	Changeover
20	1	Production
11	2	Interruption during changeover
21		Interruption during production

The next step involved removing the features that did not show a variation in value throughout the entire recording, leaving 170 features. This number of features is considerably higher than in previous studies, due to the inclusion of separate drive and tool information. In total, 52,026 data rows are available. The features consist of the following:

- Door, rapid traverse, program, and coolant status
- Feed rate, positions, position errors for each axis
- Total running time and program running time
- Program line content, number, and program path with program name
- Spindle speed
- Current, torque, modulation depth, temperature, active power, circuit voltage, speed for each axis
- Tool information

There are seven axes in this machine, namely X, Y, Z, B, C (see above), the spindle, and the tool change system. A detailed description of the features with datatype, value range, and unit can be found on GitHub^6^.

Table 7 shows the start and end timestamps of each changeover matrix.

**Table 7:** Changeover matrix timestamps.

Changeover Matrix	Starttime	Endtime
1	12.03.2024 08:16:30	12.03.2024 13:51:10
2	20.03.2024 06:43:14	20.03.2024 09:28:00
3	20.03.2024 09:28:56	20.03.2024 12:02:37
4	20.03.2024 12:02:56	22.03.2024 08:45:56
5	22.03.2024 08:45:57	22.03.2024 11:23:43

Table 8 shows all the tools used in manufacturing the three products. The last three tools were not used for production, only ‘BLUM_REINIGUNGSKOPF’ was used once to clean the manufacturing chamber due to heavy soiling.

**Table 8:** Used tools.

Tool Name	Used in Product
ENTFRAESER_D8_N_90GRAD	A, C
BOHRER_D3.3_VHM	A
SCHAFT_D8_N_AP20	B
SCHAFT_D4_N_AP20	B
ENTFRAESER_D8_90GRAD	B
PLAN_D50_N_AP4	C
SCHAFT_D12_N_AP30	C
SCHAFT_D6_N_AP15	C
3D-TASTER	A, B, C
BLUM REINIGUNGSKOPF	—
SCHAFT_D8_N_AP40	—
BOHRER_D6.8_VHM	—

## Data Records

The data is stored in one file with a recording frequency of 1s and can be found in a Zenodo repository^3^.

Product A is the last product that was produced before each changeover matrix starts. The production sequence for each changeover matrix is as follows: B, C, B, A, C, A.

The Data Records are structured as a table and stored as a CSV file, using a comma as the separator. The first two columns contain an index and timestamp formatted as YYYY-MM-DD HH:MM:SS. The following 170 columns contain all the features, with decimal points denoted by a period (‘.’). Boolean values are represented by ‘True’ and ‘False’. The last ten columns contain the labels as numerical values. Each of these label columns corresponds to a specific label set explained in Section ‘Data pre-processing’.

Table 9 contains a short description of the external material stored on GitHub^6^. A flow chart of the entire changeover process is available there, as well as the NC-code for the three manufactured products, detailed information for the used tools listed in Table 8 and a list of interruptions (see also Section ‘Data irregularities’ below) and a feature list with full description (see also Section ‘Data pre-processing’ above). A Jupyter notebook shows how to use the data set including basic pre-processing (see also Section ‘Code example’ below).

**Table 9:** List of external material.

No.	Name	Content
1	Changeover process	Detailed description of the changeover process with flow chart
2	NC-code	G-Code of the three example products
3	Tool information	Detail information on the cutting tools used for milling
4	Jupyter notebook	Example source code in Python, how to use the data set^3^
5	Data irregularities	List of interruptions
6	Feature description	List of features and description

## Technical Validation

In previous research, the authors worked on detecting sub-phases of the changeover process^10^. It turned out that the more sub-phases are detected, the worse the machine learning algorithms performed. With a growing number of sub-phases to be detected, the amount of data in the single classes for the learning process became imbalanced. Imbalance is a known problem for machine learning algorithms, therefore the imbalance of the data set^3^ will be evaluated in the next section.

The aim of the data set^3^ presented was also to improve the statistical validity by repeating the experiments. Although laboratory engineers were trained in advance, a ‘learning curve’ effect can be noticed in the data. This effect is analyzed in the related section.

### Imbalance of classes

During previous research, the authors showed that data labeling can affect the machine learning approach, as algorithms can be affected by imbalanced class counts^11^. Depending on the number of data points in the class, algorithms may not be able to accurately separate these classes from others. In the following, the word phase is used synonymously with class.

**Fig. 4:**
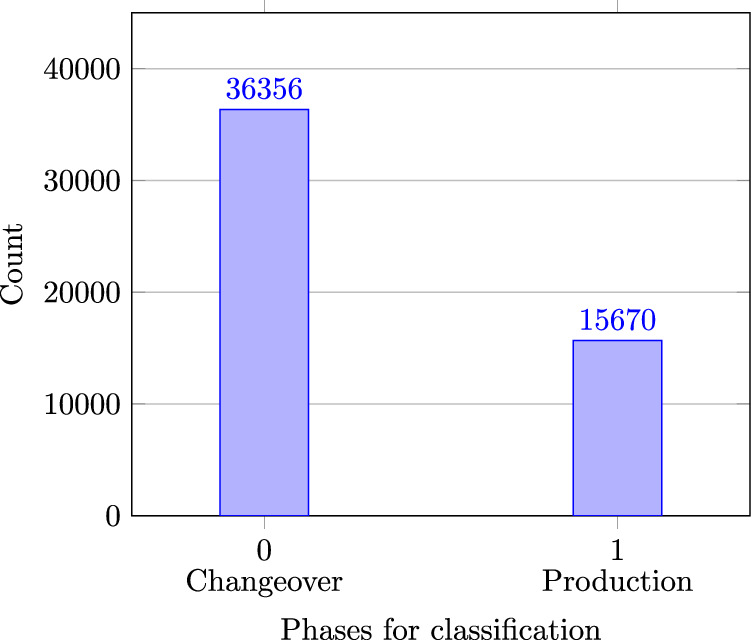
Occurrences in 2-phases (Label_03).

Figure 4 shows the frequencies of the individual phases for the approach with two changeover phases (Label_03). It can be seen that there are more than twice as many changeover phases as production phases in the data set^3^. Due to limited resources, only one series product was manufactured on the machine during the ‘Production’ phase. This is equivalent to a manufacturing order lot size of ‘1’. As machine production is deterministic, it is possible to duplicate the data points of the ‘Production’ phases depending on the desired batch size and supplement the data set^3^ accordingly. In addition to the oversampling approach described, other techniques, such as cost-sensitive classifiers, can be used^12^.

**Fig. 5:**
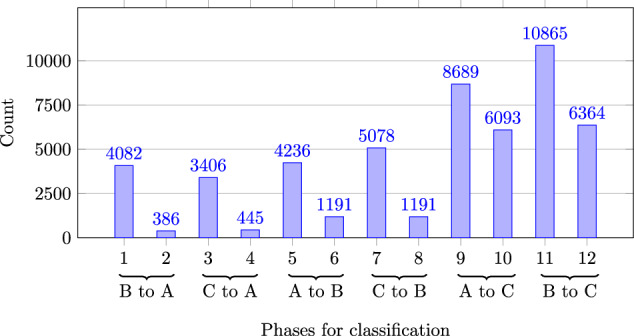
Occurences in 12-phases (Label_08).

Figure 5 shows the frequencies of the individual phases for the approach with 12 changeover phases (Label_08). It also shows that changeover phases occur more frequently than production phases (odd numbers: changeover phases, even numbers: production phases). This discrepancy is particularly clear in phases 2, 4, 6, and 8, which describe the production phases of products A and B. These are lower than the number of production phases for product C. Over all recordings, there are an average of 1,246 data points including interruptions for each production process of product C, there are 238 counts for product B and 83 occurrences for product A. Since, as described above, only one product could be manufactured after the completion of each changeover process, this number of production phases is an expected result. Another reason for the high number of changeover phases in the data set^3^ is that, as shown in Fig. 1, the ramp-up phase, i.e. the production of the first product, is considered part of the setup phase.

**Fig. 6:**
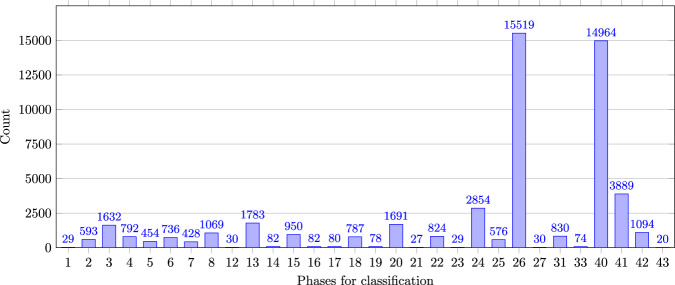
Occurences in 43-phases (Label_10).

Figure 6 shows the frequencies of the individual phases for the approach with 43 changeover phases (Label_10). Phases that are only consisting of opening and closing the door have few occurrences (Phases 1, 12, 14, 16, 17, 19, 21, 23, 43). Running the NC-code during changeover (Phase 26) and Production (Phase 40) have the most occurrences (approx. 15,000).

**Fig. 7:**
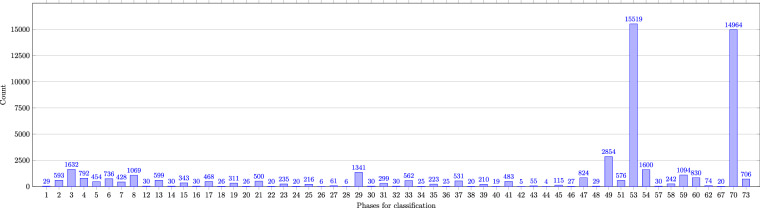
Occurences in 73-phases (Label_01).

Figure 7 shows the frequencies of the individual phases for the approach with 73 changeover phases (Label_01). As with the occurrences of the 43-phase approach (Label_10), the door opening and closing phases have few occurrences, especially phases 26 and 28 as they only occur for changeover activities where product C was produced before. This is because there are more tools needed to produce product C and all tools that are not needed for the new product are removed from the machine.

### Learning curve effect

**Fig. 8:**
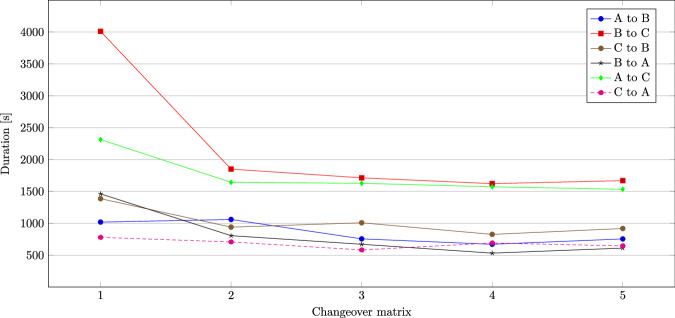
Learning curve effect.

Figure 8 shows the durations of the changeover processes for each complete changeover matrix. The durations of the individual changeover combination are shown for each changeover matrix. It can be seen that the changeover processes in changeover matrix ‘1’ consistently have the longest durations, except for the changeover process ‘A to B’. There are two possible explanations:

While executing the first changeover matrix, there was a longer interruption during the changeover from ‘B to C’, and also from ‘A to C’ strongly increasing the total durations.

As the number of repetitions of performed manufacturing operations increases, a so-called ‘learning curve’ emerges. This phenomenon, also known as ‘learning-by-doing’, describes how performance is continuously improved through repeated execution of a work step^13^ (p. 423). The term ‘experience curve effect’ is also used in the literature to describe the potential to reduce unit costs by 20 to 30 percent for every doubling of the cumulative production volume. In addition to technological advances, this is also based on learning effects that lead to increases in productivity^14^(p. 115 f.). For changeover, this means that the employee becomes increasingly familiar with the processes with each changeover process carried out, which shortens the changeover time and can extend the production time.

A reduction of changeover times from changeover matrix ‘1’ to ‘2’ can be clearly identified for the changeovers ‘C to B’ and ‘B to A’ and slightly for ‘C to A’ (see Fig. 8). Despite prior instruction and training, the laboratory engineer was not yet fully familiar with the procedures. One reason may be that, unlike experienced industrial workers, laboratory engineers at universities do not carry out changeover activities daily.

If the data set^3^ is to be used for modeling changeover times, the authors recommend not using the first and possibly also the second changeover matrix or correcting the data set using interpolation techniques. As the ‘learning curve effect’ is relevant and known, the relevant data was left unchanged in the data set^3^.

As all interruptions are labeled in the data set^3^, it is also possible to exclude interruptions during pre-processing. Please see Label_06, and Label_07 and the corresponding description of data irregularities in the GitHub^6^ material.

## Usage Notes

### License

The data set^3^ is available on the Zenodo platform. External material is available on the GitHub platform^6^. The license for the data set^3^ and all external material is Creative Commons Attribution 4.0 International^15^. Researchers are free to share and adapt the presented data set, but they must give credit to the authors with a reference, provide a link to the license, and indicate changes to the original data set^3^ and other additional material.

### Application examples

In previous research, the authors have worked on different application examples with an older data set^7^ (see Table 1):

An application example is forecasting the energy demand of basic G-commands from the NC-code. Here, Latin Hypercube Sampling is used as an efficient method of Design of Experiments to train a machine learning model for the forecasting^16^.

Another example of the application of the data set^3^ is the automatic detection of human changeover activity from the NC data. No additional manual feedback from the operator is needed. Various machine learning model were analyzed in their ability to detect changeovers accurately. It was also analyzed if sub-phases of the changeover process can be detected only from the machine’s NC data. Different machine learning techniques were evaluated, like random forests or neural networks^10^.

This automatic changeover detection can also be accomplished by applying time series machine learning techniques to classify phases of the machine changeover^17^.

Although the authors applied an existing data set^7^, the application examples are valid for the new data set^3^ as well. The authors are currently working on validating the previous research results with the new data set^3^. The application example of classifying Label_03 with machine learning was chosen as a code example applying the new data set^3^ (please see Section ‘Code example’).

### Data irregularities

Although thoroughly planned and executed, even in laboratory production, deviations from the optimal manufacturing process can occur. Deviations usually occur in the production process where people are involved. In the case of this sample production, this is the changeover process. The subsequent production process, on the other hand, runs in the milling machine using a deterministic NC program and is not subject to human influence if the NC program has been tested in advance and is within verified machine production parameters.

The changeover processes of the machine for the production of the three products were summarized and abstracted in a standard changeover process. A description of this changeover standard is published on GitHub^6^. The faults in the setup process were documented, and the data points were clearly labeled accordingly. The documentation of the individual deviations and the names of the corresponding labels are also uploaded to GitHub^6^.

As deviations from planned procedures also occur in series production, where they are also seen as a source of potential improvements^18^, it was decided to leave the faults in the data set^3^. It is recommended that the user either correct the deviations or filter them out of the data set^3^ using the corresponding labels.

### Code example

The Jupyter notebook included in the external materials serves as an example of applying machine learning techniques to the data set^3^. Initially, the data is imported, and the boolean and string columns are converted into numerical formats. Missing values are replaced by zeros. Subsequently, the data set^3^ is partitioned into training and testing subsets and standardized. The final step involves training a Random Forest model and assessing its performance on the test set^6^.

## Data Availability

The Python code for simple preprocessing and training a Random Forest can be found in a Jupyter notebook on GitHub: https://github.com/ElMoe/Production-Data-Set-for-Five-Axis-CNC-Milling-with-Multiple-Changeovers.
